# Irisflorentin improves α-synuclein accumulation and attenuates 6-OHDA-induced dopaminergic neuron degeneration, implication for Parkinson’s disease therapy

**DOI:** 10.7603/s40681-015-0004-y

**Published:** 2015-02-02

**Authors:** Yue-Mi Chen, Shih-Ping Liu, Hsin-Lien Lin, Ming-Chia Chan, Yen-Chuan Chen, Yu-Ling Huang, Min-Chen Tsai, Ru-Huei Fu

**Affiliations:** 1 Graduate Institute of Immunology, China Medical University, 404 Taichung, Taiwan; 2 Center for Neuropsychiatry, China Medical University Hospital, 404 No. 91, Hsueh-shih Road, Taichung, Taiwan; 3 Graduate Institute of Basic Medical Science, China Medical University, 404 Taichung, Taiwan

**Keywords:** Irisflorentin;, Parkinson’s disease;, *Caenorhabditis elegans;*, Dopaminergic neurons;, α-Synuclein

## Abstract

Parkinson’s disease (PD) is a degenerative disorder of the central nervous system that is characterized by progressive loss of dopaminergic neurons in the substantia nigra pars compacta as well as motor impairment. Aggregation of α-synuclein in neuronal cells plays a key role in this disease. At present, therapeutics for PD provides moderate symptomatic benefits, but it is not able to delay the development of the disease. Current efforts toward the treatment of PD are to identify new drugs that slow or arrest the progressive course of PD by interfering with a disease-specific pathogenetic process in PD patients. Irisflorentin derived from the roots of *Belamcanda chinensis* (L.) DC. is an herb which has been used for the treatment of inflammatory disorders in traditional Chinese medicine. The purpose of the present study was to assess the potential for irisflorentin to ameliorate PD in *Caenorhabditis elegans* models. Our data reveal that irisflorentin prevents α-synuclein accumulation in the transgenic *Caenorhabditis elegans* model and also improves dopaminergic neuron degeneration, food-sensing behavior, and life-span in a 6-hydroxydopamine-induced Caenorhabditis elegans model, thus indicating its potential as a anti-parkinsonian drug candidate. Irisflorentin may exert its effects by promoting *rpn-3* expression to enhance the activity of proteasomes and down-regulating egl-1 expression to block apoptosis pathways. These findings encourage further investigation on irisflorentin as a possible potent agent for PD treatment.

## 1. Introduction

Parkinson’s disease (PD) is one of the most common neurodegenerative disorders and is becoming increasingly prevalent among elderly populations. PD is seen not only as a major health problem, but since it is care-intensive, it also carries a significant economic challenge. PD impairs the motor control of the body and is the result of the selective death of dopaminergic (DA) neurons in the substantia nigra of the midbrain [1]. The disease is characterized by the aggregation of α-synuclein to form Lewy bodies in the neuron [2]. The cause of most cases of PD (sporadic) remains unclear and likely results from a complex interplay of genetic and environmental factors [3]. Only 5-10% of PD cases (familial) occur because of the mutation of several specific genes. These genes include α-synuclein (SNCA), parkin (PRKN), leucine-rich repeat kinase 2 (LRRK2), PTEN-induced putative kinase 1 (PINK1), DJ-1 and ATP13A2 [4].

α-synuclein is a small acidic protein and is found mainly at the presynaptic terminals of neurons. It can be divided into the N-terminal α-helical domain, the central hydrophobic domain, and the acidic carboxyl-terminal domain [2]. α-synuclein may maintain the supply of synaptic vesicles in neuronal terminals and regulate dopamine release to control voluntary and involuntary movements [5]. Aggregation of α-synuclein has been observed in both sporadic and familial forms of PD. Three mutations in the α-helical domain (A53T, A30P, E46K) were indicated to promote oligomer/fibril formation and were associated with autosomal dominant early onset PD [6].

Many reports have shown a link between toxin exposure and raised risk of PD [7]. The 6-hydroxydopamine (6-OHDA) is a specific neurotoxin which targets catecholamine neurons through the dopamine active transporter (DAT). When 6-OHDA is injected into the median forebrain bundle or into the neostriatum of brain, it causes an irreversible loss of DA neurons in the ventral midbrain. The consistent loss of dopamine innervation in target areas is linked with a range of long-term, behavioural deficits.

**Fig. 1:**
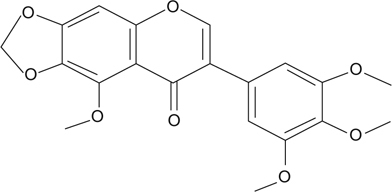
Chemical structure of irisflorentin.

Thus, a 6-OHDA –induced lesion is the most widely used animal model of PD [8].

Presently there is no effective therapy for PD. Current drugs, including levodopa, dopamine agonists, and monoamine oxidase B inhibitors, only relieve and delay the symptoms [9]. The most hopeful therapeutic direction for PD involves the discovery of small compounds that can prevent the disease as well as improve its symptoms. A number of Chinese herbs have been confirmed to be effective neuroprotective agents. *Belamcanda chinensis* (L.) DC. (synonym: *Iris domestica*) is a flowering perennial herb distributed over the region of East Asia, and is valuable in Chinese villages for its medicinal uses. The dried rhizome (Shegan in Chinese) has been listed for treatments such as pharyngitis, coughing, swollen spleen, asthma and liver, gonorrhea, malaria, and arrow poisoning [10]. Isoflavones were the main bioactive constituents of Belamcandae Rhizoma [11], and have physiological benefits including anti-oxidant, anti-inflammatory, anti-cancer, and hypoglycemic properties [12]. Irisflorentin (Fig. 1) is the major isoflavone component derived from Belamcandae Rhizoma [13]. Here, we consider that irisflorentin could be tested as a prophylactic as well as an adjuvant agent for its advantageous effects on PD using *C. elegans* model system. We also address the possible mechanisms of irisflorentin action.

## 2. Materials and methods

### 2.1. Strains, maintenance, and synchronization

*C. elegans* of wild-type Bristol N2, transgenic BZ555 (Pdat-1::GFP; green fluorescent protein [GFP] visible in dopaminergic neurons) and transgenic OW13 (Punc-54::α-synuclein::YFP+unc-119; human α-synuclein protein with yellow fluorescent protein [YFP] observable in the muscles) were gained from the Caenorhabditis Genetics Center (University of Minnesota). On the basis of previous standard procedures [14], we maintained the animals on NGM plates seeded with the *E. coli* strain OP50 as food sources at 22°C. Fertilized eggs were collected by hypochlorite treatment of gravid adults. After 20 h incubation at 22°C in M9 buffer to acquire synchronized L1 larvae, the animals were spread on OP50/NGM plates and then incubated for 24 h at 22°C to obtain L3 larvae.

### 2.2. Food clearance assay

Synthesized irisflorentin (mol. wt. 386.35, 98% purity) was purchased from Fusol Material Co., Ltd. (Tainan, Taiwan), and then dissolved in dimethyl sulfoxide (DMSO) to 1M as a master stock solution. A food clearance assay was used to determine the effect of irisflorentin on *C. elegans* physiology [15]. *E. coli* were cultured overnight and then re-suspended at a final OD of 6.6 in nematode S-medium. Irisflorentin was diluted into the E. coli suspension to the desired concentrations. Fifty microliters of the final mixture was added per well in a 96-well plate. Approximately 20-30 synchronized L1 animals in 10 μl of S-medium were added to an *E. coli* suspension containing a series of concentrations of irisflorentin and incubated in a 96-well microtiter plate at 25°C. The absorbance (OD 595 nm) of the culture was measured every day for 6 days using a SpectraMax M2 Microplate Reader (Molecular Devices, Silicon Valley, CA).

### 2.3. Quantitative analysis of α-synuclein aggregation

Aggregation of α-synuclein protein was assessed in control and irisflorentin- treated OW13 animals. Synchronized OW13 L3 larvae were cultured on OP50/NGM plates containing 0.04 mg/mL FUDR (Sigma, St. Louis, MI) and irisflorentin for 72 h at 22°C, then washed three times with M9 buffer and transferred to 2% agarose pads on glass slides, mounted with 100 mM sodium azide and enclosed with a coverslip. Immobilized animals were imaged on an Axio Observer inverted fluorescence microscope (Carl Zeiss, Germany) to monitor the aggregation of α-synuclein protein, and the aggregation was quantified using AxioVision software by measuring fluorescence intensity.

### 2.4. Exposure to 6-OHDA and treatment with irisflorentin

In brief, 50 mM 6-OHDA and 10 mM ascorbic acid were added to OP50/S-medium mix with irisflorentin. Synchronized L3 larvae were then transferred onto the treated cultures, incubated for 1 h at 22°C and mixed gently every 10 min. After 1 h of treatment, animals were washed three times with M9 buffer and then incubated in OP50/NGM plates with irisflorentin. After 24 h, animals were transferred to OP50/NGM plates containing irisflorentin and 0.04 mg/mL 5-fluoro-2′-deoxyuridine (FUDR, Sigma, St. Louis, MI) to lessen the production of progeny. Animals were scored with various assays 72 h after treatment.

### 2.5. Quantitative analysis of dopaminergic neurodegeneration

Analysis of dopaminergic neurodegeneration was performed in animals treated with 6-OHDA or irisflorentin/6-OHDA, as described previously. After 72 h of treatment at 22°C, BZ555 animals were washed three times using M9 buffer, and then mounted onto a 2% agar pad on a glass slide using 100 mM sodium azide (Sigma, St. Louis, MI) and enclosed with a coverslip. Imaging of immobilized animals was carried out with an Axio Observer inverted fluorescence microscope (Carl Zeiss MicroImaging GmbH, Göttingen Germany). Fluorescence intensity was quantified using AxioVision software (Carl Zeiss, Göttingen, Germany).

### 2.6. Analysis of food-sensing behavior

Briefly, test plates were prepared by spreading *E. coli* overnight at 37°C in a ring with an inner diameter of 1 cm and an outer diameter of 8 cm on 9-cm diameter NGM agar plates to prevent the animals from reaching the edge of the plate during the test. Well-fed 6-OHDA-treated or irisflorentin/6-OHDA-treated adult animals were washed with M9 buffer and then transferred to the center of a test plate with or without a bacterial lawn in a drop of M9 buffer. Five minutes after the transfer, the locomotory rate of each animal was counted in 20-s intervals. The slowing rate was calculated as the percentage of the locomotory rate in the bacteria lawn compared with that in the no-bacteria lawn. The average slowing rate among 10 animals was defined as the result of each analysis. In all tests, plates were numbered so that the experimenter was blind to the treatment of the animal.

**Table 1:** List of primers used for qPCR assays.

**Proteasome subunits**		
	**Forward (5’ → 3’)**	**Reverse (5’ → 3’)**
*pas-1*	GGCTGATCTCAACCAGTATTACACA	GAACAAAAGAGCACATCCCAAAC
*pas-2*	CGGCCGTAATGCAGGAATAT	AAGAAGCGATGCTCCAAACG
*pas-3*	CGGAGAGGAAATGCCAGTTG	ACGGTCTCTTTCCTCCAATCTG
*pas-4*	GTCGTACCACCAGGATCACAAA	TGCTGCAGCTCATCATTAACTTTT
*pas-5*	CAACATATTGGCGTCACATTCG	TGCCCGTTCGACCAGAGT
*pas-6*	CGAGAAATCAACTCCGGAACA	AAGTGTGTCGCGAAGAGCAA
*pas-7*	TTTCCAAGTCGAGTACGCTCAA	TTGCCACGAATTGCAATCAT
*pbs-1*	TCAGCACTGGAACCACTCTCA	TCGGTTCCGACGACAACTC
*pbs-2*	ATTTTGGAGCGTGATTTTAAGGTT	GGCGCGTTGGACAAGCT
*pbs-3*	GCTCCACGCGATTTCGTT	GCGCCAGAAGTTTTCACAAAC
*pbs-4*	GGGCAACAGCCGTACTTGTT	CGATCCATAATGGCATAGCAGAA
*pbs-5*	CTGCAATTTGTGCCACATCAC	TCACGTCCATTGGTGGAAGA
*pbs-6*	GATATGAGCGTCCGGAACTCA	ACGGAACGAATCCTTCATCAA
*pbs-7*	CTCTACGCCAAACGTTGCAA	ACTCCGGCGACAACAAGTG
*rpt-1*	TGGAAACATCAAGGTGCTTATGG	CTCATGAGAGCGGGATCGA
*rpt-2*	CCTGACGCCGCTAGCAAA	GCAACTTCAGACGGCATCTG
*rpt-3*	TGGAGAAGGACCACGAATGG	GATGGGCTGTTTTCCTTTGC
*rpt-4*	GTCAAGTTGTCCGACGGATTC	TGGCAAACATTCCAGCTTCTG
*rpt-5*	GAAGATGAATGTCAACAAGGATGTAAA	TGCATTGTGCTCCGTTGAAG
*rpt-6*	CCGAAGAATCCGATGAGAAAAC	CACTTTTTGCTGCGCATCA
*rpn-1*	CGGAAAGCCAAAGACAATCAC	GAGATATTCATCGTTCGCCAACT
*rpn-2*	TGACATTGTTGAACAGATGGAGATC	TGCGGCTGCGTTTGAA
*rpn-3*	ATACATTGTGGCGAAGGCTATTG	TGTACCGAGGTCCATCACGAA
*rpn-5*	GGAGAGCACAACATGCGTATGA	CAGCGAGACGTTCGAAAGTG
*rpn-6*	AATATTGGAAAAGCACCTGAAATGT	TTTGATGTGGAAGTGAAGTCATTGT
*rpn-7*	TCATTCAGTTGGCCGCTCTT	TGTGGCGATAGATAGCGATCAA
*rpn-8*	TCAGGAAGTTCACGATGATGGA	TCTGAAGGCACATGCTCGAA
*rpn-9*	GGGTGCAGCCAAGAGTTTTAGA	GGAGTTGACATCGTTCCTCCAT
*rpn-10*	AGTACTATGATTTGTGTCGACAATTCG	GGAGCCGAGTTGGTTGGAA
*rpn-11*	ACGTTTTCGCTATGCCACAGT	TGGATCGACCGCTTCGA
*rpn-12*	CAAAGGAGCCAAAAGATCTTGTC	CACTGAGAACCTTCGTCAACTCA
**Apoptosis mediators**		
	**Forward (5’ → 3’)**	**Reverse (5’ → 3’)**
*egl-1*	CTAGCAGCAATGTGCGATGAC	GGAAGCATGGGCCGAGTAG
*ced-9*	TGCTCAGGACTTGCCATCAC	TTGACTCTCCGATGGACATTCTT
*ced-4*	AAGTCGAGGATTAGTCGGTGTTG	AGAGCCATTGCGAGTGACTTG
*ced-3*	TCAACGCGGCAAATGCT	GCCTGCACAAAAACGATTTTC
House keeping genes		
	**Forward (5’ → 3’)**	**Reverse (5’ → 3’)**
*cdc-42*	CTGCTGGACAGGAAGATTACG	CTCGGACATTCTCGAATGAAG
*pmp-3*	GTTCCCGTGTTCATCACTCAT	ACACCGTCGAGAAGCTGTAGA
*Y45F10D.4*	GTCGCTTCAAATCAGTTCAGC	GTTCTTGTCAAGTGATCCGACA

### 2.7. RNA isolation and quantitative RT–PCR

Total RNA was extracted from synchronized control or experimental adult animals using TRIzol reagent (Invitrogen, Carlsbad, CA) according to the manufacturer’s instructions. cDNA was generated using the SuperScript One-Step RT-PCR system (Invitrogen, Carlsbad, CA). SYBR Green real-time qPCR analyses were carried out with a 1:20 dilution of cDNA using an ABI StepOnePlus system (Applied Biosystems). Data were calculated with the comparative 2ΔΔC_t_ method using the geometric mean of *cdc-42, pmp-3* and *Y45F10D.4* as the endogenous control [16]. Table 1 shows details of the primers used for this study.

### 2.8. 26S proteasome activity assays

Briefly, using a Precellys 24 homogenizer (Bertin Technologies, Montigny-le-Bretonneux, France), animals were lysed in a proteasome activity assay buffer containing 50 mM Tris-HCl (pH 7.5), 250 mM sucrose, 5 mM MgCl_2_, 2 mM ATP, 1 mM dithiothreitol and 0.5 mM EDTA. The lysate was centrifuged at 10,000 *g* for 15 min at 4°C. For each experiment, 25 μg of total lysate was loaded into each well of a 96-well microtiter plate, after which a fluorogenic substrate was added. For testing the chymotrypsinlike activity of the proteasome, Z-Gly-Gly-Leu-AMC (Enzo Life Sciences, Farmingdale, NY) was used as a substrate. After incubation for 1 h at 25°C, fluorescence (an excitation wavelength of 380 nm and an emission wavelength of 460 nm) was detected with a SpectraMax M2 Microplate Reader (Molecular Devices, Silicon Valley, CA).

### 2.9. Statistical analysis

Statistical analyses are expressed as mean ± standard deviation (SD) from independent tests. Three replicates were done of each test. The differences between two means were determined by a student’s *t*-test. Values of *P* < 0.05 were determined to be statistically significant.

## 3. Results

### 3.1. Determining the irisflorentin concentration range for C. elegans treatment by food clearance assay

**Fig. 2:**
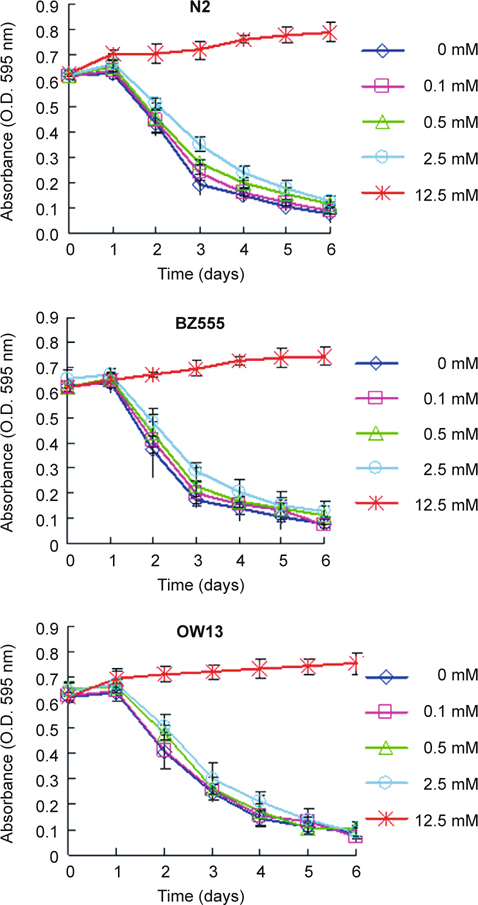
The concentration of irisflorentin for experiments was determined with a food clearance assay. Between 20 and 30 newly hatched L1 synchronized animals of N2, BZ555 or OW13 were incubated in *E. coli* (OD A_595_ = 0.6) in a 96-well plate at 25°C containing different irisflorentin concentrations to a total volume of 60 μL. The OD of the plate was measured daily for 6 days. Note the lower OD (~0.6) in a well due to the decreased path length of the 60 μL final suspension compared to the 1 cm path length in a spectrophotometer. The OD of *E. coli* was recorded daily for each concentration of irisflorentin.

To assess the effects of irisflorentin on DA neuron degeneration and α-synuclein accumulation, we first determined the optimal concentrations of irisflorentin to evaluate in our *C. elegans* PD models via food clearance assay. Given the advantage of the short life cycle and the ability of *C. elegans* to grow in a liquid culture of *E. coli*, irisflorentin was tested at the rate at which the *E. coli* suspension was consumed. Each adult animal can produce hundreds of offspring that quickly consume the limited *E. coli* supply. Consequently, the OD of the wells without irisflorentin was significantly reduced in 3 days in N2, BZ555 and OW13 strains (Fig. 2). The addition of 0.1 mM, 0.5 mM or 2.5 mM irisflorentin to the cultures containing N2, BZ555 or OW13 strains revealed no effect on food clearance compared to that in the control animals, whereas animals exposed to 12.5 mM irisflorentin had considerably delayed food clearance (Fig. 2). Additionally, animals exposed to 12.5 mM irisflorentin did not produce offspring over the time course of the experiment (data not shown), which was linked to the lack of clearance of the *E. coli* source. In the experiments following this assay, animals were treated with irisflorentin at concentrations of up to 2.5 mM.

### 3.2. Irisflorentin decreased accumulation of α-synuclein protein

**Fig. 3:**
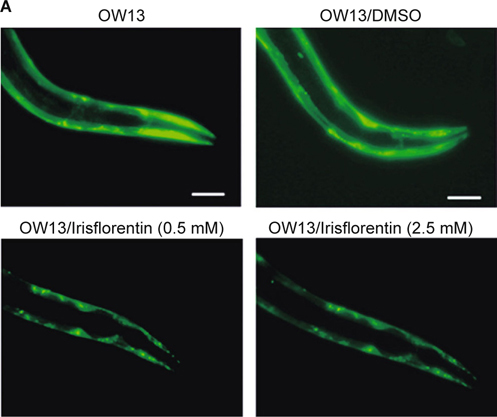

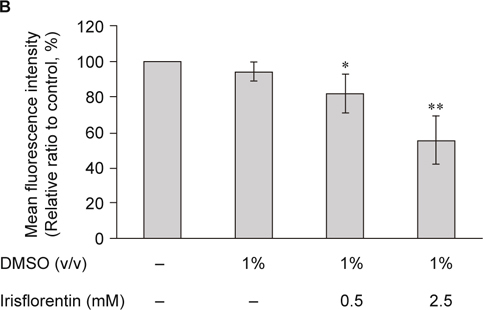
Irisflorentin hinders α-synuclein accumulation in the OW13 strain of *C. elegans*. (A) YFP expression patterns in muscles of transgenic *C. elegans* strain OW13. The figures show fluorescence images. Scale bar, 50 μm. (B) Graphical representation for fluorescence intensity of YFP expression pattern in muscles of transgenic *C. elegans* strain OW13 as quantified using AxioVision software. The data represent the mean ± SD (n = 10). An asterisk (^*^) indicates significant differences between the control samples and the irisflorentintreated samples (^*^ *P* < 0.05, ^**^ *P* < 0.01).

*C. elegans* lacks orthologous genes of α-synuclein. However, the genetic flexibility of the animal allows transgenic expression of human α-synuclein genes and the study of α-synuclein aggregation. OW13 animals of untreated and irisflorentin- treated groups were observed for their α-synuclein protein accumulation range. Treatment of animals with irisflorentin displayed significantly diminished fluorescence intensity of accumulation compared to that of untreated animals (Fig. 3A). Irisflorentin reduced the YFP expression of OW13 animals in a dose-dependent manner. At 2.5 mM irisflorentin, the fluorescence intensity of YFP expression related to α-synuclein protein accumulation in OW13 animals decreased by about 45% (*P* < 0.01) compared to that in untreated animals (Fig. 3B).

### 3.3. 6-OHDA-induced degeneration of DA neurons was diminished by treatment of irisflorentin

**Fig. 4:**
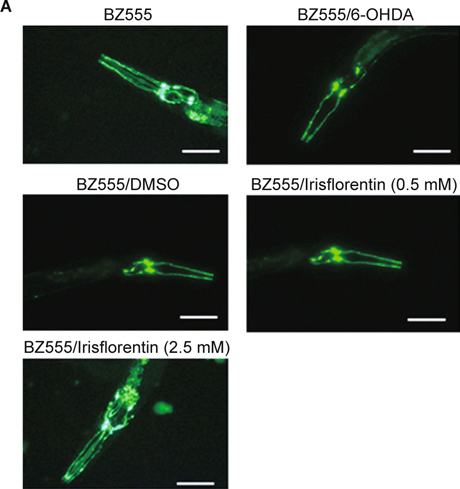

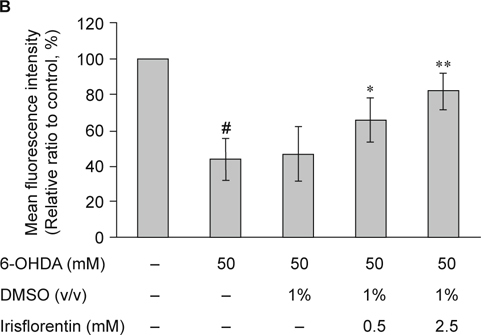
Irisflorentin protects dopaminergic (DA) neurons of *C. elegans* from degeneration resulting from 6-OHDA treatment. (A) GFP expression pattern in DA neurons of transgenic *C. elegans* strain BZ555. The figures show the fluorescence images. Scale bar, 50 μm. (B) Graphical representation for fluorescence intensity of GFP expression pattern in DA neurons of a transgenic *C. elegans* strain as quantified using AxioVision software. The data represent the mean ± SD (n = 10). A hash (#) indicates significant differences between 6-OHDA-treated and untreated animals (*P* < 0.001); an asterisk (^*^) indicates significant differences between the 6-OHDA-treated control samples and the Irisflorentin/6-OHDA-treated samples (^*^ *P* < 0.05, ^**^ *P* < 0.01).

*C. elegans* contains eight DA neurons, including one pair of anterior deirid (ADE) neurons and two pairs of cephalic (CEP) neurons in the head position, and one pair of posterior deirid (PDE) neurons in the posterior lateral region. Selective degeneration of these DA neurons was observed through exposure to 6-OHDA. To examine irisflorentin efficacy, we assessed neuronal viability by testing the loss of expression of a GFP reporter construct in DA neurons of 6-OHDA-treated BZ555 animals. Data showed that ADE and CEP neurons lost a partial GFP with a slight reduction in GFP expression in PDE neurons after 6-OHDA treatment (Fig. 4A). When animals were treated with irisflorentin, remarkable protection was found in DA neurons with ADE, and CEP neurons were presenting an augmented expression of GFP (Fig 4A). We further calculated the fluorescence intensity in DA neurons using AxioVision software. In 6-OHDA-treated animals, the mean fluorescence (GFP) intensity lessened by about 56% (*P* < 0.01) compared to that of untreated animals (Fig. 4B). Irisflorentin increased the GFP expression in a dose-dependent manner. At 2.5 mM irisflorentin, the fluorescence intensity of GFP expression in DA neurons of 6-OHDA-treated animals increased by about 1.9-fold (*P* < 0.01) compared to that in animals treated only with 6-OHDA (Fig. 4B).

**Fig. 5:**
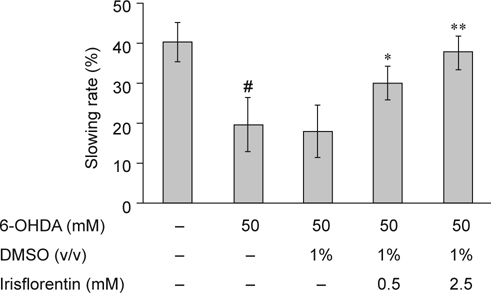
Irisflorentin improved food-sensing behavior in 6- OHDA-treated N2 *C. elegans*. The locomotory rate (frequency of bending) of 6-OHDA-untreated animals, 6-OHDA-treated animals, or irisflorentin/6-OHDA-treated animals with or without bacteria lawns was tested. Shown are the slowing rates calculated as the percentage reduction of the locomotory rate in the bacteria lawn as compared with that in no bacteria lawn. The data represent the mean ± SD (n = 20). A hash (#) indicates significant differences between 6-OHDA-treated and untreated animals (*P* < 0.01); an asterisk (^*^) indicates significant differences between the 6-OHDA-treated control samples and the irisflorentin/6-OHDA-treated samples (^*^ *P* < 0.05, ^**^ *P* < 0.01).

### 3.4. Irisflorentin improved food-sensing behavior of 6-OHDA-treated C. elegans

It has been revealed that 6-OHDA-treated animals show a phenotype of DA neuron degeneration that should affect food-sensing behavior [17, 18]. *C. elegans* bend their bodies to move themselves for migration, and the rate of movement is determined by the bending frequency. Once animals come across food, they lessen the bending frequency to feed themselves more effectively. 6-OHDA-treated animals, however, fail to display a decremental bending frequency in response to food sensing. Therefore, the function of DA neurotransmission in *C. elegans* is linked with this food-sensing behavior. We examined whether 6-OHDA treatment in *C. elegans* induces a defect in this function in 3-day-old animals that were synchronized for age. Wild-type N2 animals presented a 40% reduction in bending frequency upon contact with bacteria (Fig. 5). In contrast, 6-OHDA-treated animals revealed a significant lessening in this decremental response compared with wild-type N2 animals (20%, *P* < 0.01). Irisflorentin improved the decremental response of 6-OHDA-treated animals in a dosedependent manner. At 2.5 mM irisflorentin treatment, 6-OHDAtreated animals presented decreased bending movements upon contact with bacteria by about 1.9-fold (*P* < 0.01) compared with that in untreated animals (Fig. 5).

### 3.5. Irisflorentin raised somatic proteasome activity by enhancing proteasome regulatory subunit rpn-3 expressionin an α-synuclein-expressing model of C. elegans

**Fig. 6:**
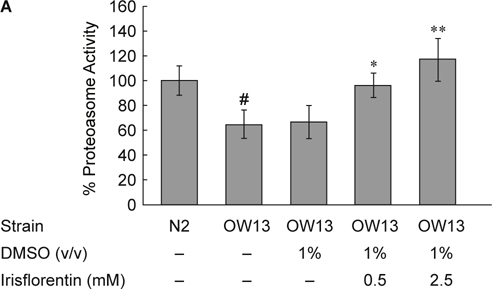

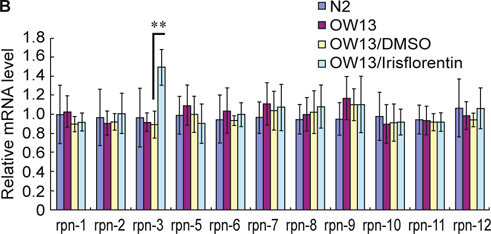
Irisflorentin increases proteasome activity by enhancing *rpn-3* expression in the OW13 strain of *C. elegans*. (A) Chymotrypsin-like activity of the proteasome was monitored by Z-Gly-Gly-Leu-AMC digestion in a day 3 extract of adult animal containing equal amounts of total protein. The data represent the mean ± SD (n = 3). A hash (#) indicates significant differences between N2 and OW13 animals (*P* < 0.01); an asterisk (^*^) indicates significant differences between the OW13 control samples and the irisflorentin-treated OW13 samples (^*^ *P* < 0.05, ^**^ *P* < 0.01). (B) Irisflorentin raises the expression of rpn3 of the regulatory subunit of proteasome in the OW13 strain of *C. elegans*. Quantitative real-time RT-PCR experiments show the expression level of the Rpn subunit of the 26S proteasome using cDNAs isolated from OW13 control or irisflorentin-treated animals. The data represent the mean ± SD (n = 3). An asterisk (^*^) indicates significant differences between the OW13 control samples and the irisflorentintreated samples (^**^ *P* < 0.01).

We hypothesized that the ubiquitin proteasome system might be changed in irisflorentin-treated OW13 animals. To evaluate whether the observed decrease in total α-synuclein in the muscle of OW13 animals was the result of augmented proteasomal activity, we analyzed 26S proteasome activity upon treatment with irisflorentin by using a proteasome activity assay with a fluorescent substrate. As shown in Fig. 6A, the basal level of chymotrypsin-like proteasome activity was about 35% lower in OW13 animals compared to that in N2 animals (*P* < 0.01). Irisflorentin treatment significantly augmented the chymotrypsin-like proteasome activity in OW13 animals in a dose-dependent manner. Chymotrypsin-like proteasome activity following 2.5 mM irisflorentin treatment was augmented by about 1.8-fold in the OW13 animals (*P* < 0.01) (Fig. 6A). These results indicate that enhanced proteasome activity results in a reduction in α-synuclein and that irisflorentin treatment can improve proteasome activity in the animal model of PD. We therefore next assessed whether the proteasome activity of irisflorentin-treated OW13 animals correlated with an augmented expression level of the regulatory particles of the 19S proteasome or the catalytically active subunits of the 20S proteasome. The basal level of all subunits was not different in OW13 animals compared to that in N2 animals. Irisflorentin enhanced the expression level of the *rpn-3* of the regulatory subunit. The expression level of rpn-3 following 2.5 mM irisflorentin treatment was augmented by about 67% in the OW13 animals (*P* < 0.01) (Fig. 6B).

**Fig. 7:**
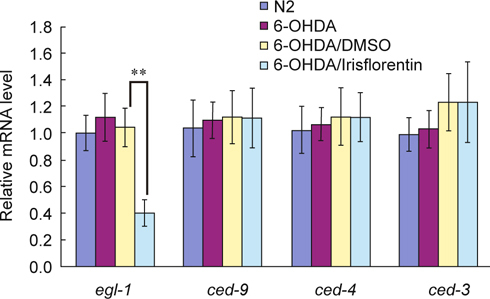
Irisflorentin reduces *egl-1* expression in apoptosis modulation in 6-OHDA-treated *C. elegans*. Quantitative real-time RT-PCR experiments show the expression levels of *egl-1, ced-3, ced-4* and *ced-9* using cDNAs isolated from N2, 6- OHDA-treated, or irisflorentin/6-OHDA-treated animals. The data represent the mean ± SD (n = 3). An asterisk (^*^) indicates significant differences between the 6-OHDA-treated control samples and the irisflorentin/6-OHDA-treated samples (^**^ *P* < 0.01).

### 3.6. Irisflorentin diminished apoptosis modulator egl-1 expression in 6-OHDA- treated C. elegans

We hypothesized that an important aspect of the apoptosis pathway might be changed in the 6-OHDA-treated animals by irisflorentin. To evaluate whether the observed decrease in DA neuron degeneration of *C. elegans* was the result of reduced apoptosis activity, after treating the animals with irisflorentin, we used a real-time PCR method to analyze the mRNA levels of *egl-1, ced-3, ced-4* and *ced-9*, which are related to apoptosis of *C. elegans*. As shown in Fig. 7, the expression level of *egl-1, ced-3, ced-4* and *ced-9* was not augmented in 6-OHDA-treated animals compared to that in untreated animals. At 2.5 mM irisflorentin, the expression level of egl-1 in 6-OHDA-treated animals lessened by about 64% (*P* < 0.01) compared to that in animals treated only with 6-OHDA (Fig. 7).

## 4. Discussion

In this study, our methods used the benefits of the *C. elegans* model for drug testing, including convenient and exact visualization of live DA neurons and α-synuclein accumulation. These tests could be powerful for inexpensive, rapid examination and screening of drugs for PD. Our data demonstrate that irisflorentin attenuates α-synuclein accumulation; reduces DA neuron degeneration; and recovers food-sensing behavior in a pharmacological or transgenic *C. elegans* model. To the best of our knowledge, this is the first report of the anti-parkinsonian role of irisflorentin in an animal model.

The *SNCA* gene (*PARK1*) encodes human α-synuclein. In PD, the accumulation of α-synuclein is a particular challenge for DA neurons, especially as they aggregate in inclusions and aggresomes and overwhelm the cellular machinery for their degradation. These effects are possibly involved in the PD-related dysregulation of chaperones, a down-regulation of the degradation system itself, and an accelerating loss in overall cellular homeostasis [19]. Irisflorentin diminished the accumulation of α-synuclein, thus arresting its toxic effect in the cells. We identified that the preventive effect of irisflorentin was due to its regulation of the proteosome activity. It is well known that the ubiquitin-proteosome system salvages the cells from harm under stressful conditions [20].

The 26S proteasome complex of *C. elegans* is composed of a 20S “catalytic core” and 19S “regulatory caps”. 19S can be further separated into two distinct substructures containing a ring of six homologous ATPase subunits of the AAA family (Rpt-1-6), two α-helical solenoid structures (Rpn-1 and Rpn-2), eight essential RP non-ATPase subunits (Rpn-3, Rpn-5-9 and Rpn-11-12), and two Ub receptors (Rpn-10) [21]. The effect of irisflorentin may be linked with augmented expression of *rpn3*, a subunit of the 19S proteasome. Rpn-3 expression is tightly controlled; both overexpression and deletion of Rpn-3 impair proteasome functions. The exact mechanisms underlying these results require further study [22].

Given that DA neurons are more susceptible to oxidative stress, reactive oxygen species are important regulators in neuron apoptosis and death. 6-OHDA destroys neuronal cells by generating reactive oxygen species such as the superoxide radical [23]. The neuroprotective role of irisflorentin in 6-OHDA-induced DA neuron degeneration and food-sensing behavior defects is possibly related to its antioxidant and antiapoptotic activity. Additionally, it has also been demonstrated that mitochondrial dysfunction induced by 6-OHDA triggers the release of cytochrome c and the activation of caspase-3 [24]. Caspase-3 is a crucial effector in apoptosis that is triggered through different pathways in various mammalian cell types, especially in the cytochrome c-dependent apoptosis pathways. In *C. elegans*, the BH3-only domain protein EGL-1, the Apaf-1 homolog CED-4, and the CED-3 caspase are involved in apoptosis triggering, whereas the Bcl-2 homolog CED-9 inhibits apoptosis. CED-9 is obligatory to block CED-4 by abrogating CED-4 accumulation in the perinuclear space in response to proapoptotic stimuli. EGL-1 antagonizes CED-9, causing CED-4 oligomerization and triggering apoptosis through CED-3 caspase activation [25]. In this study, we showed that irisflorentin lessened the expression of *egl-1*, which, in turn, antagonized 6-OHDA-induced DA neuron apoptosis. The protective effects of irisflorentin on DA neurons could possibly be connected with these events; but the mechanistic aspects need to be studied in more detail.

Evidence implies that chronic neuroinflammation is linked with the pathophysiology of PD [26]. Activation of microglia and augmented levels of pro-inflammatory mediators such as TNF-α, IL-1β and IL-6 have been described in the substantia nigra of PD patients and in animal models of PD [27]. It is hypothesized that activated microglia secretes high levels of proinflammatory cytokines that impair neurons and further activate microglia, resulting in further inducing of inflammation and neurodegeneration. Moreover, DA neurons are more susceptible to proinflammatory mediators than other cell types. In previous research, irisflorentin was found to suppress LPS-induced inflammatory responses in RAW 264.7 macrophages [28]. It markedly decreases the transcriptional and translational levels of inducible nitric oxide synthase as well as the production of NO, and downregulates expression levels of TNF-α, IL-1β and IL-6. These effects act via ERK1/2 - and p38-mediated activator protein-1. Hence, irisflorentin may also be able to improve inflammation in the brains of patients with PD and lessen DA neuron damage.

The present study shows that irisflorentin is a new member on the list of isoflavones with anti-parkinsonian properties and provides traditional Chinese medicine practitioners with information about the utilization of herbs containing irisflorentin in the treatment of neuron-related diseases. This readily obtainable compound provides a convenient, low-cost, and highly effective means of regulating the survival and function of DA neurons. In the future, we plan to investigate the precise mechanism by which irisflorentin maintains DA neuron activity in other animal PD models and evaluate the suitability of irisflorentin for disease control in clinical trials.

### Acknowledgements

This work was supported by the Ministry of Science and Technology (Taiwan) (MOST 103-2314-B-039-025), the Taiwan Ministry of Health and Welfare Clinical Trial and Research Center of Excellence (MOHW103-TDU-B-212-113002) and China Medical University (DMR-104-052).

### Declaration of interest

The authors declare no conflicts of interest for this work.
